# P28 Profile of uropathogens and trend of their antimicrobial resistance at a trauma care facility: a 10 year analysis

**DOI:** 10.1093/jacamr/dlad077.032

**Published:** 2023-08-02

**Authors:** Aparna Ningombam, Arpan Kumar Thakur, Smriti Srivastava, M Nizam Ahmed, Madhavi Kirti, Rajesh Malhotra, Purva Mathur

**Affiliations:** Department of Laboratory Medicine, JPN Apex Trauma Centre, AIIMS, New Delhi, India; Department of Laboratory Medicine, JPN Apex Trauma Centre, AIIMS, New Delhi, India; Department of Microbiology, AIIMS, New Delhi, India; Department of Microbiology, AIIMS, New Delhi, India; Department of Microbiology, AIIMS, New Delhi, India; Department of Orthopaedics, AIIMS, New Delhi, India; Department of Laboratory Medicine, JPN Apex Trauma Centre, AIIMS, New Delhi, India

## Abstract

**Background:**

Urinary tract infections (UTIs) are one of the most common healthcare-associated infections (HAIs). Trauma patients acquire UTIs almost exclusively due to hospital or treatment-related causes. Healthcare-associated UTIs (HA-UTIs) have increased morbidity, antimicrobial treatment cost and length of stay. Temporal evaluation of HA-UTIs will help in assessing the actual magnitude of the problem and the variation in antibiograms. In this study, we have evaluated the prevalence of uropathogens and their antimicrobial resistance (AMR) pattern over 10 years from patients admitted to a level-1 trauma centre in India.

**Methods:**

The study reports the pathogen profile and their antibiograms from the period 2012 to 2022. Antibiotic susceptibility was done in our laboratory using Vitek 2. In cases where the CLSI guidelines do not recommend Vitek 2, disc diffusion testing was done. The most prevalent organisms with their antimicrobial susceptibility testing were analysed using appropriate statistical measures.

**Results:**

From a total of 3432 isolates, the most common uropathogens were *Escherichia coli* (1074), *Klebsiella pneumoniae* (643) and *Pseudomonas aeruginosa* (638). Amongst Gram-positives, *Enterococcus faecium* (171) was the most significant. The AMR pattern of the Gram-negative organisms showed that the majority were MDR. Amikacin resistance was consistently in the range of 20%–25% for all *E. coli* isolates except in 2021 and 2022, where it was 8.47% and 17.82%. *K. pneumoniae* isolates (>60%) were consistently resistant to amikacin, cefepime, ciprofloxacin and piperacillin/tazobactam. However, there was a slight dip in resistance to amikacin in 2017, which was 52.38%. Imipenem showed consistent resistance in the 30%–60% range, but there has been a spike in resistance (>60%) after the pandemic. Resistance to ceftazidime, cefepime, ciprofloxacin and amikacin was >80% for *Pseudomonas* isolates except in 2019 and 2020, where their resistance pattern was within 60%–80%. Imipenem resistance was >60% throughout the period except for 2021, where resistance was 53.3% (8/15).

**Conclusions:**

All the antibiotics showed an upward trend in resistance post the pandemic except amikacin in isolates of *E. coli.* In conclusion, AMR trends are very important, and antibiograms should be revised on a timely basis.

